# Walking Biomechanics and Spine Loading in Patients With Symptomatic Lumbar Spinal Stenosis

**DOI:** 10.3389/fbioe.2021.751155

**Published:** 2021-11-18

**Authors:** Seyed Javad Mousavi, Andrew C. Lynch, Brett T. Allaire, Andrew P. White, Dennis E. Anderson

**Affiliations:** ^1^ Department of Orthopaedic Surgery, Beth Israel Deaconess Medical Center, Boston, MA, United States; ^2^ Department of Orthopaedic Surgery, Harvard Medical School, Boston, MA, United States

**Keywords:** lumbar spinal stenosis, trunk posture, spine motion, compressive loading, optoelectronic motion capture, gait

## Abstract

Symptomatic lumbar spinal stenosis is a leading cause of pain and mobility limitation in older adults. It is clinically believed that patients with lumbar spinal stenosis adopt a flexed trunk posture or bend forward and alter their gait pattern to improve tolerance for walking. However, a biomechanical assessment of spine posture and motion during walking is broadly lacking in these patients. The purpose of this study was to evaluate lumbar spine and pelvic sagittal angles and lumbar spine compressive loads in standing and walking and to determine the effect of pain and neurogenic claudication symptoms in patients with symptomatic lumbar spinal stenosis. Seven participants with symptomatic lumbar spinal stenosis, aged 44–82, underwent a 3D opto-electronic motion analysis during standing and walking trials in asymptomatic and symptomatic states. Passive reflective marker clusters (four markers each) were attached to participants at T1, L1, and S2 levels of the spine, with additional reflective markers at other spinal levels, as well as the head, pelvis, and extremities. Whole-body motion data was collected during standing and walking trials in asymptomatic and symptomatic states. The results showed that the spine was slightly flexed during walking, but this was not affected by symptoms. Pelvic tilt was not different when symptoms were present, but suggests a possible effect of more forward tilt in both standing (*p* = 0.052) and walking (*p* = 0.075). Lumbar spine loading during symptomatic walking was increased by an average of 7% over asymptomatic walking (*p* = 0.001). Our results did not show increased spine flexion (adopting a trunk-flexed posture) and only indicate a trend for a small forward shift of the pelvis during both symptomatic walking and standing. This suggests that provocation of symptoms in these patients does not markedly affect their normal gait kinematics. The finding of increased spine loading with provocation of symptoms supports our hypothesis that spine loading plays a role in limiting walking function in patients with lumbar spinal stenosis, but additional work is needed to understand the biomechanical cause of this increase.

## Introduction

Lumbar spinal stenosis (LSS) is a common degenerative spinal condition with the prevalence of 19%–47% in adults over age 60, depending on the criteria used. Lumbar spinal stenosis is symptomatic in 10%–14% of the adult population, and its prevalence and associated health and economic consequences are expected to increase with the aging of the population (Katz and Harris, 2008; Kalichman et al., 2009; Ishimoto et al., 2012). The most common symptom attributed to LSS is neurogenic claudication characterized by pain and discomfort radiating from the spine to the legs along with sensory loss, fatigue, weakness, and balance problems (Katz and Harris, 2008; Suri et al., 2010). Limited tolerance for standing and walking is characteristic of symptomatic LSS and is the leading cause of disability and restricted mobility, and it is also the most frequent indication for spinal surgery, in patients over 65 years old (Deyo et al., 2010; Hijikata et al., 2020). LSS symptoms are often initiated or provoked by walking or prolonged standing, particularly when the lumbar spine is in extended (lordotic or upright) postures, and gradually aggravated to the point that the patient stops walking. Trunk flexion or bending forward can partially relieve the symptoms by reducing the magnitude of lumbar lordosis, increasing spinal canal diameter, and decompressing the nerves (Katz and Harris, 2008). Therefore, it is clinically believed that patients with LSS adopt a flexed (hunched) trunk posture or bend forward and alter their gait pattern to improve tolerance for walking (Katz and Harris, 2008). While these clinical observations are the basis for some of the therapeutic exercises and clinical recommendations to increase walking capacity in patients with LSS, they have not yet been scientifically tested and quantified.

A biomechanical assessment of spine posture and motion during walking is broadly lacking in patients with LSS, and the available results are not consistent (Toosizadeh et al., 2015; Wang et al., 2021). To the authors’ knowledge and a recently published systematic review (Wang et al., 2021), only three studies investigated spine kinematics (postural angles) in patients with LSS during walking, and two of them reported kinetic variables including hip and knee flexion moments and paravertebral muscle activities (Kuwahara et al., 2016; Goto et al., 2017; Igawa et al., 2018). The study of Goto et al. (2017) is the only one that measured the spine flexion angle of five men and one woman with LSS during the beginning of treadmill walking and when leg symptoms appeared. Thoracic and pelvic angles (reflecting the absolute movement in space) were increased after walking, but the spine angle reflecting the relative movement between the thorax and pelvis did not significantly change when symptoms appeared.

The purpose of this study was to evaluate trunk posture, particularly lumbar spine and pelvis angles, and lumbar spine compressive loads in standing and walking and to determine the effect of pain and neurogenic claudication symptoms, in patients with symptomatic lumbar spinal stenosis. Optoelectronic motion analysis along with detailed musculoskeletal modeling have been recently implemented in healthy and patient populations to measure spine posture and motion and estimate spine loading during walking and activities of daily living (Schmid et al., 2016; Mousavi et al., 2018; Burkhart et al., 2020). Here, we utilize this methodology to characterize lumbar spine posture, pelvic tilt, and spine loading in patients with LSS during standing and walking and to determine whether these parameters change following provocation of neurogenic claudication symptoms.

We hypothesized that patients would display an increased trunk flexion posture and spine loading during walking and in the presence of claudication symptoms.

## Methods

### Subjects

Seven participants aged 44–82, with symptomatic LSS confirmed by imaging and clinical examination, who were scheduled for spine decompression surgery (laminectomy with or without fusion) for lumbar spinal stenosis were recruited. Characteristics of the participants (four women and three men) are presented in Table 1. These were the mean ± SD of age: 64.4 ± 13.8 years, height: 164 ± 9.5 cm, body mass: 79 ± 29.8 kg, and BMI: 29.2 ± 3.8 kg/m^2^. Participants were excluded if they had conditions (unrelated to LSS) that altered walking or spine function, such as history of traumatic spinal injury or surgery, vascular insufficiency, Parkinson’s disease, stroke, or cognitive impairment. The study was approved by the Institutional Review Board of Beth Israel Deaconess Medical Center, and all patients provided written informed consent prior to participation.

**TABLE 1:** Characteristics of the participants.

Participants	Age	Sex	Height (cm)	Weight (kg)	BMI (kg/m^2^)	Surgery level	Pain at rest^a^	Pain after walking^a^	Walking capacity time (min)
1	50	F	166.3	73.3	26.5	L3–S1	3	6	30
2	82	F	158.9	79.9	31.7	L2–L5	8	10	2.4
3	73	M	170.6	100.5	34.5	L3–L5	3	6	15
4	44	M	172.7	72.5	24.3	L5–S1	2	4	17
5	66	F	149.8	71.1	31.7	L3–L5	5	7	3.8
6	75	F	155.8	62	25.5	L4–L5	1	2	1.7
7	61	M	175.2	93.4	30.4	L5–S1	3	8	1.7

a Based on the Brief Pain Inventory (BPI) at rest and after walking capacity test. Zero (0) denotes no pain and 10 denotes the worst pain.

### Experimental Procedure

All patients underwent a 3D opto-electronic motion analysis during standing and walking trials between 2 and 10 days before surgery. Passive reflective marker clusters (four markers each) were attached to participants at T1, L1, and S2 levels of the spine, with additional reflective markers at other thoracic and lumbar spinal levels, as well as the head, manubrium of the sternum, posterior superior iliac spines, shoulders, upper and lower arms and legs, and feet. The marker position was recorded by a motion analysis system (Vicon Motion Systems, Centennial, CO).

### Tasks

Whole-body motion data was collected in asymptomatic and symptomatic states. Asymptomatic state refers to the state or time that participants did not experience any neurogenic claudication symptoms. Almost all of the participants experienced a range of back and/or leg pain during the relaxed sitting position, but they were able to distinguish this pain from the neurogenic claudication symptoms that are usually provoked during walking and forced them to stop or limit their walking. To produce the symptomatic state, participants performed a standard walking capacity test, walking over ground or on a motorized treadmill at a self-selected pace until reporting the onset of neurogenic claudication symptoms, up to a maximum of 30 min (Rainville et al., 2012). Time to onset of symptoms and distance walked were recorded. Participants reported their pain severity both before and after provocation based on the 10 Brief Pain Inventory. The following tasks were conducted in a consecutive order (Table 2): 1) static upright standing posture (asymptomatic), 2) walking at a self-selected pace without neurogenic claudication symptoms present (asymptomatic) (three trials), 3) walking after onset of neurogenic claudication symptoms (symptomatic) (three trials), and 4) static upright standing posture (symptomatic).

**TABLE 2:** Testing procedure and outcome measurements.

**Asymptomatic state**	**Walking capacity test to provoke symptoms**	**Symptomatic state**
Trunk posture		Trunk posture
Spine and hip motion		Spine and hip motion
Spine loading		Spine loading

### Data Processing and Musculoskeletal Modeling

**FIGURE 1:**
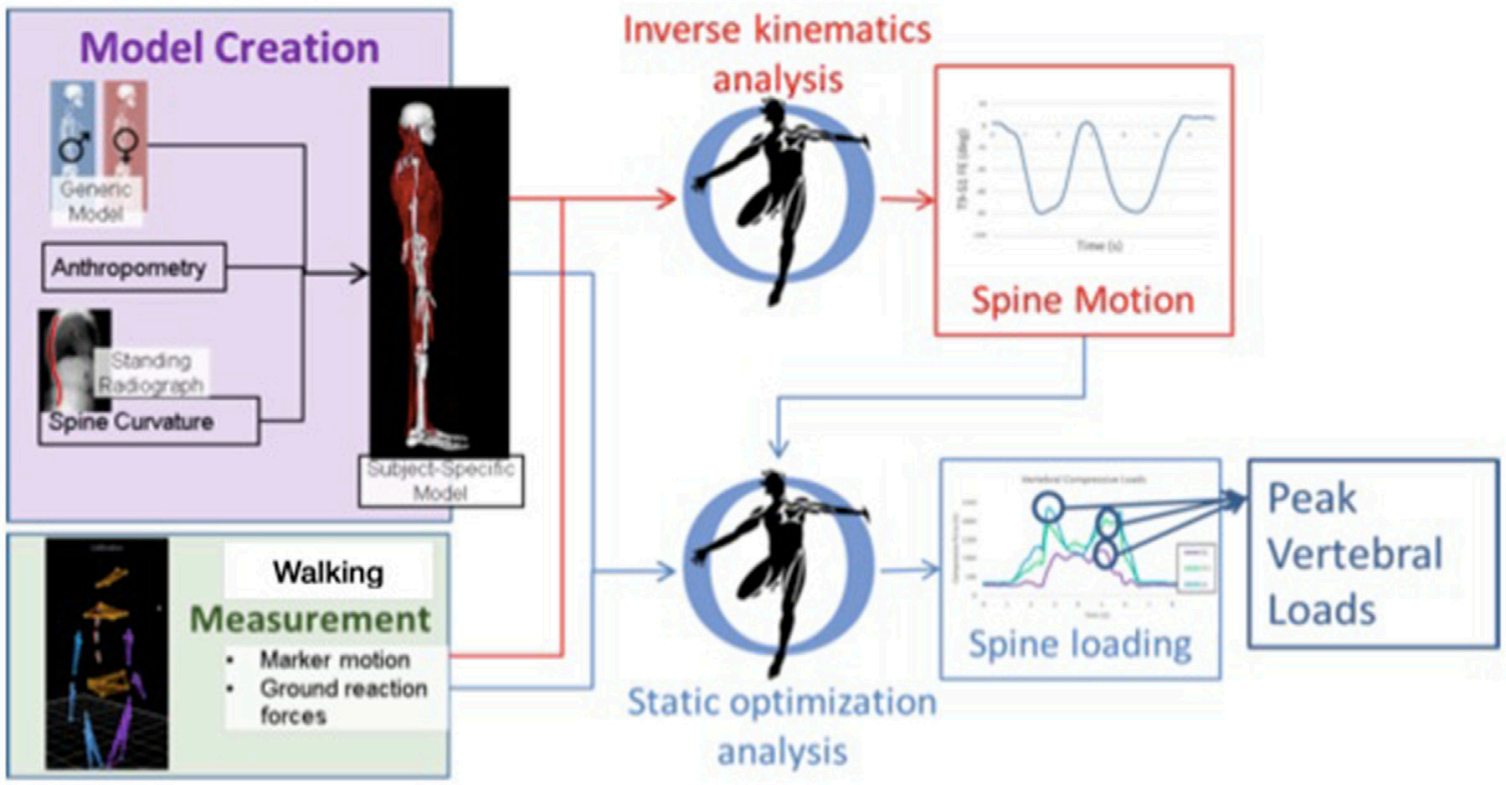
Basic workflow for creating subject-specific musculoskeletal model to determine spine motion and loading. Walking trials were measured, and outcomes of body motion and loading were evaluated for a single gait cycle in each trial. Spine loading was evaluated during standing and walking trials.

A whole-body musculoskeletal model was created for each participant, incorporated with our established model of the thoracolumbar spine, and adjusted according to patient age, sex, height, weight, motion analysis measurements, and standing spine radiographs (Bruno et al., 2015; Bruno et al., 2017; Burkhart et al., 2020) (Figure 1). Base model was first adjusted according to anthropometrics and marker data in a neutral posture using the OpenSim scale tool (Delp et al., 2007). Lumbar spine curvature was assessed from the subject’s pre-treatment standing radiograph (available from the online medical records); thoracic curvature (Cobb angle) was estimated based on our recently proposed regression equation using the participant’s thoracic angle calculated from spine markers, age, and BMI, and intervertebral angles in the model were adjusted accordingly producing a subject-specific model (Hashemirad et al., 2013; Grindle et al., 2020). Measured marker data for standing and walking trials were applied to the subject-specific model to estimate movements of the spine and other body joints. Similar to prior studies, we applied kinematic constraints to limit spinal degrees of freedom when evaluating spinal motion (Actis et al., 2018; Ignasiak et al., 2018; Beaucage-Gauvreau et al., 2019; Alemi et al., 2021). We allowed six spinal degrees of freedom in our models, two each in flexion-extension, lateral bending, and axial rotation, which produces realistic, repeatable spine motions from motion analysis data with relatively low marker error (Alemi et al., 2021). With this, the flexion-extension motion of the spine has two independent coordinates applied to sections T1–T9 and T9–S1, respectively, and the reported spine flexion outcome in this study is the flexion of the T9–S1 segment of the spine (Alemi et al., 2021). An inverse kinematics analysis was performed to evaluate body positions during standing and walking, including lumbar flexion/extension angles and pelvic anterior/posterior tilt. Kinematics were applied in a static optimization analysis to solve for muscle and joint loads, and thereby lumbar spine compressive loading, during standing and walking trials. The magnitude of peak compressive load within each subject was evaluated as the average of the peak load at all lumbar vertebral levels. The outcomes of pelvic tilt, spine flexion, pelvic tilt plus spine flexion, and lumbar compressive load were then averaged across one gait cycle. Postural outcomes were referenced to the asymptomatic neutral standing trial. Secondary outcomes of peak angle and ROM of the hips, pelvis, and spine during walking were also evaluated.

### Statistical Analysis

Mixed-effects regression analysis was used to examine the effects of walking and symptoms on the outcome measures, with participant as a random effect. Lumbar load was analyzed similarly for the effects of walking and symptoms.

## Results

**FIGURE 2:**
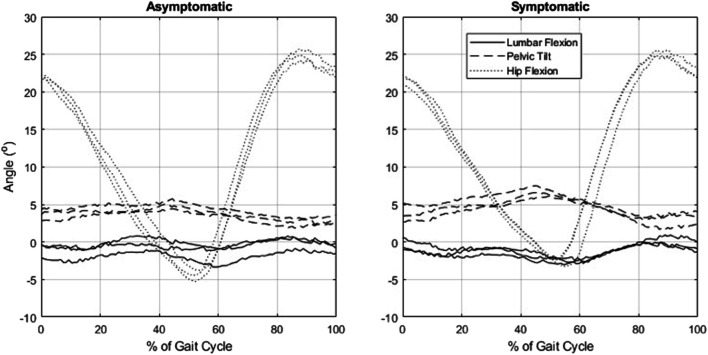
Example data from a participant with symptomatic lumbar spinal stenosis showing sagittal plane pelvic, spinal, and hip kinematics during a single gait cycle in three independent trials, as evaluated by optical motion capture and inverse kinematics analysis.

**FIGURE 3:**
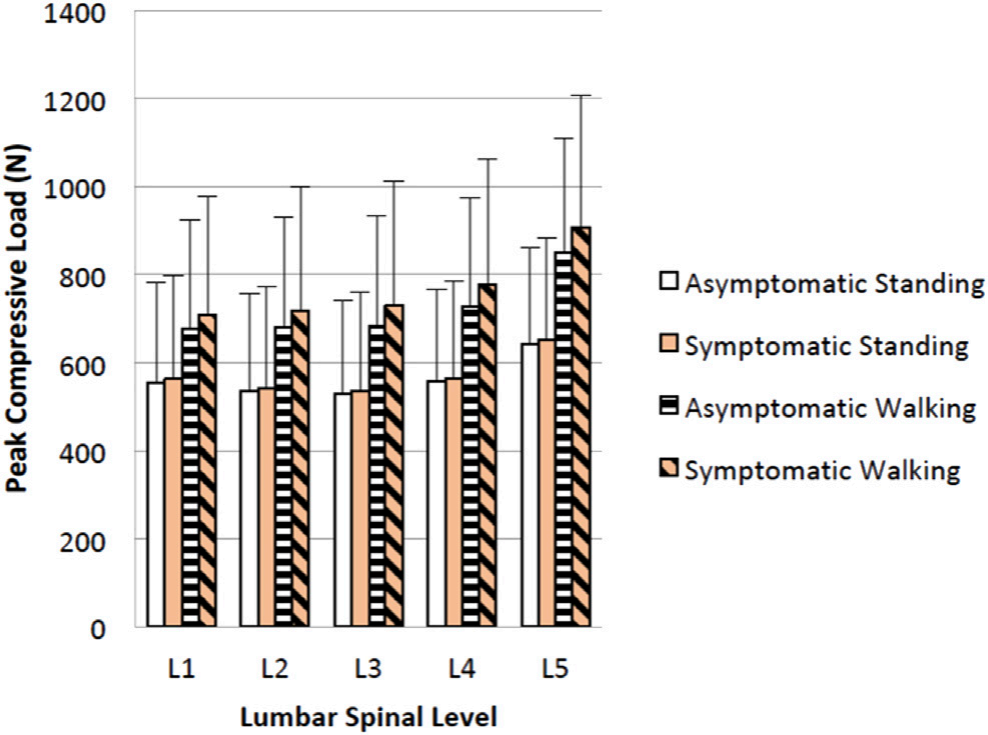
Mean (SD) of peak lumbar loads for asymptomatic and symptomatic standing and walking.

Participants walked for an average of 10.0 min (range 1.7–30.0 min) to provoke symptoms and reported an average increase in pain by 2.6 points (range 1–5), from 3.57 to 6.14 (*p* < 0.05) (Table 1). Mean (SD) of the lumbar spine flexion, forward pelvic tilt (pelvic flexion), and spine flexion + pelvic tilt angles were 3.4° (3.4°), 0.7° (4.5°), and 4.1° (2.6°), respectively, in asymptomatic walking and 3.4° (2.6°), 1.2° (4.3°), and 4.6° (3.2°), respectively, in symptomatic walking (Table 3). Example kinematics data from a single participant during a single gait cycle in three independent trials is presented in Figure 2. The spine was slightly flexed during walking, but this was not affected by symptoms. Pelvic tilt was not different when symptoms were present, but suggests a possible effect of more forward tilt in both standing (average change 1.1°, *p* = 0.052) and walking (average change 0.5°, *p* = 0.075). Provocation of symptoms did not affect the peak angle or ROM of the hips, pelvis, or spine during walking (Table 3). Lumbar loading averaged 564 (217) N in asymptomatic standing and was increased by an average of 26% during asymptomatic walking. Loading in symptomatic standing was not larger than asymptomatic standing, while loading during symptomatic walking (769 ± 269) was increased by an average of 7% over asymptomatic walking (704 ± 221) (*p* = 0.001). Figure 3 shows peak compressive loading of each lumbar level in standing and walking.

**TABLE 3:** Mean (SD) of average postural measurements (forward pelvic tilt, spinal flexion, and pelvic tilt + spinal flexion) relative to asymptomatic neutral standing, in asymptomatic walking, symptomatic walking, and symptomatic neutral standing.

	Asymptomatic walking	Symptomatic walking	Symptomatic neutral standing
Pelvic tilt (°)	0.7 (4.5)	1.2 (4.3)^c^	1.1 (1.2)^b^
Spine flexion (°)	3.4 (3.4)^a^	3.4 (2.6)^a^	−1.4 (2.9)
Pelvic tilt + spine flexion (°)	4.1 (2.6)^a^	4.6 (3.2)^a^	−0.3 (2.6)

a Different than 0 (*p* < 0.05).

b Different than 0 (*p* = 0.052).

c Different than asymptomatic walking (*p* = 0.075).

## Discussion

**FIGURE 4:**
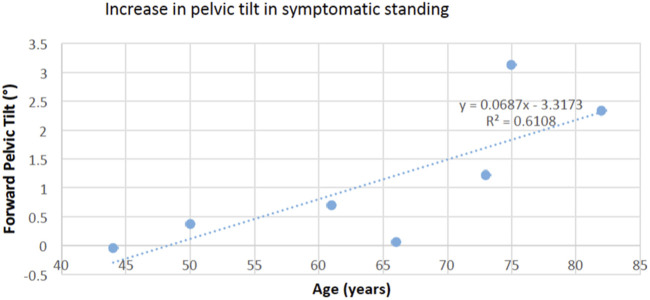
The increase in forward pelvic tilt in symptomatic standing was positively associated with age (*p* = 0.038), suggesting a larger effect in older adults.

Our results did not show increased spine flexion (adopting a trunk-flexed posture) and only indicate a trend for a small forward shift of the pelvis during both symptomatic walking and standing. This suggests that provocation of symptoms in patients with symptomatic LSS does not markedly affect their normal gait kinematics and does not support our overall hypothesis. Our results are in line with Goto et al. (2017) who reported increased thoracic and pelvic sagittal plane angles, but no change in spine flexion angle, immediately after the symptoms appeared. While bending forward during clinical examination can relieve pain and symptoms in patients with LSS, our results are not in line with the clinical observations that patients with LSS bend forward or adopt a stooped posture during walking to improve tolerance for walking, by relieving pressure on the nerves (Katz and Harris, 2008). We also noticed that forward pelvic tilt (pelvic flexion) when symptoms are present was associated with age in standing position (Figure 4), but spine flexion and loading was not. This suggests that the effects of LSS symptoms may not be uniform, but dependent on patient characteristics. A recent motion analysis study on patients with LSS showed that the patients adopt two different strategies during walking; some of them used a trunk-flexed posture to increase step length and hip extension angle, while others walked with upright trunk posture to decrease step length and hip extension angle (Igawa et al., 2018). Both of these patterns were attributed to patients’ efforts to decrease the activation of psoas major muscles and therefore decrease the degree of lumbar lordosis during walking, but the study did not compare kinematics with and without symptoms (Igawa et al., 2018). A recent standing radiographic study also showed that patients with LSS with mild to moderate spinopelvic deformity [defined as 10° or more difference between pelvic incidence (PI) and lumbar lordosis (LL) angles, also called PI-LL mismatch] chose a trunk-flexed strategy, but patients with moderate to severe deformity adopted a more upright posture (Buckland et al., 2016). Finally, while adopting a trunk-flexed posture strategy might be temporarily effective in reducing symptoms in some patients, walking with this position is posturally unstable and energy inefficient, as it demands compensatory motions and higher muscular activity to maintain dynamic balance (Saha et al., 2007; Saha et al., 2008). This may soon lead to general or local muscular fatigue, forcing patients to stop or limit their walking.

Our results suggest an increase in lumbar spinal compressive loading when the neurogenic claudication symptoms were provoked. Loads on the spine cannot be measured directly, although musculoskeletal modeling can be used to estimate spinal loading given appropriate measurements of body motion. This is the first study to estimate the magnitude of lumbar spine loading during walking in asymptomatic and symptomatic states in patients with LSS. The increased lumbar compressive loading may be partially explained by the observed changes in the overall trunk kinematics in symptomatic state, though these changes were not statistically significant. However, other possible changes in spine and lower extremity kinematics and kinetics could also lead to increased loading, such as increased non-sagittal motions, increased dynamic variability of trunk motion (or sway), or increased ground reaction forces. Future analyses are needed to explore these possibilities to identify the mechanisms by which spine loading is increased in these patients.

While compelling research supports the link between higher spine loading and increased risk of spinal tissue injury and back pain (van Dieën et al., 2009), it is also plausible that increased spine loading may aggravate symptoms and decrease walking capacity in patients with LSS by reducing the size of the spinal canal and dural sac cross-section and diameter or increasing epidural pressure. This assumption can be supported by imaging studies that reported a reduction of the dural sac cross-sectional area in weight-bearing standing position compared to supine position, which was associated with increased severity of symptoms and decreased walking capacity in patients with LSS (Kanno et al., 2012; Lau et al., 2017). In addition, loading and unloading the spine through a weight vest or vertical traction harness in LSS patients while walking on a treadmill resulted in shorter and longer time for appearance of symptoms and total walking time, respectively (Oğuz et al., 2007). Our results show that spine loading increases by an average of 48 N with symptoms, while Oğuz et al. (2007) reported that wearing a weighted vest of 10 kg, or approximately 98 N, reduced total walking time in LSS patients by about 25%. Thus, the increased loading seen here with symptoms is of a magnitude that is likely to significantly impact walking performance in this population.

Physical therapy plays a central role in treatment of LSS symptoms with generally low effectiveness, though the evidence is limited and not consistent (Ammendolia et al., 2013; Schneider et al., 2019). In addition, the current therapeutic exercises do not specifically target underlying biomechanical and neuromuscular factors behind symptom provocation or mobility limitation. Decompression surgery with or without fusion can directly address the underlying pathology of nerve compression, and once the pressure on the nerves is released, tolerance for walking reliably improves (Katz and Harris, 2008; Weinstein et al., 2010; Fritsch et al., 2017). However, approximately one third of the patients are not satisfied with the postoperative outcomes, mainly in terms of residual pain and poor function (Weinstein et al., 2010; Rainville et al., 2012; Fritsch et al., 2017). Therefore, future biomechanical studies are required to assess how gait and posture change with surgical or rehabilitative treatments and whether these changes can contribute to the post-treatment improvement in patient outcomes and walking capacity (Toosizadeh et al., 2015; Goto et al., 2017; Wang et al., 2021).

We acknowledge the small sample size as a limitation of this study that may limit generalizability of the findings. However, the repeated-measure nature of the analyses reduces the impact of the small sample size, and the evaluation of walking biomechanics before and after provocation of symptoms is a novel aspect and strength of this study. While evaluation of spine loading is another strength of this study, the use of musculoskeletal models has a number of associated limitations. Spine loading estimates are not very sensitive to cost function in a standard optimization approach (Arjmand and Shirazi-Adl, 2006), as used here, but a limitation of standard optimization is that it does not accurately predict antagonistic muscle activations, which occur in a variety of trunk loading conditions (Granata and Marras, 1995; Granata et al., 2005) and could play an important role in patients with LSS. Electromyography-assisted or double-linear optimization approaches could be used in future studies to address this limitation and improve predictions of spine loading during walking (Li and Chow, 2020). Overall, additional studies are needed to alleviate these shortcomings and to determine the effects of rehabilitation and surgical treatments on spine loading and postural outcomes.

## Conclusion

In patients with LSS, spinal flexion was not increased after provocation of symptoms, which does not support the hypothesis and commonly held assumption that patients adopt flexed spine postures to increase spinal canal diameter and decompress the nerves, thereby relieving or delaying symptoms. A biomechanical analysis showed that spine loading increased in the symptomatic state, supporting the idea that spine loading, symptoms, and walking limitations are all interconnected. Additional studies of walking and spine biomechanics in this population are needed to better understand this issue (Suda et al., 2002; Comer et al., 2010).

## Data Availability

The original contributions presented in the study are included in the article/supplementary material, and further inquiries can be directed to the corresponding author.
